# Soluble FGL2, a novel effector molecule of activated hepatic stellate cells, regulates T-cell function in cirrhotic patients with hepatocellular carcinoma

**DOI:** 10.1007/s12072-014-9568-y

**Published:** 2014-09-25

**Authors:** Ying Sun, Dong Xi, Wen Ding, Faxi Wang, Haili Zhou, Qin Ning

**Affiliations:** Department of Infectious Disease, Tongji Hospital of Tongji Medical College, Huazhong University of Science and Technology, No 1095, Jiefang Avenue, Wuhan, 430030 China

**Keywords:** Soluble FGL2 protein, Hepatic stellate cells, LX2 cells, Immune regulation, T cells, HCC

## Abstract

**Purpose:**

To investigate the effects of soluble FGL2 (sFGL2) secreted by hepatic stellate cells (HSCs) on immune suppression in cirrhotic patients with hepatocellular carcinoma (HCC).

**Methods:**

Serum sFGL2 levels were examined by ELISA in 40 patients with HCC, liver cirrhosis (LC) or chronic HBV (CHB) infection. A double staining of the immunofluorescence analysis of α-SMA and FGL2 was performed in two cirrhotic liver specimens. The expression of FGL2 in the LX2 cell line was analyzed by immunofluorescence, Western blot and flow cytometry. T-cells purified from HCC patients using magnetic beads were cultured with LX2 cells at different ratios with anti-CD3-stimulating or FGL2-blocking antibodies. The proliferation index (PI) of CD8 + T cells was assessed by flow cytometry, and the secretion of IFN-γ was measured by ELISA.

**Results:**

sFGL2 levels are significantly higher in patients with HCC or LC compared with those with CHB (*p* = 0.0039/*p* = 0.0020). Among HCC patients, those with cirrhosis exhibited significantly higher levels of sFGL2 compared with non-cirrhotic individuals (*p* = 0.0108). The expressions of FGL2 and α-SMA overlapped in HSCs in liver specimens. FGL2 protein secreted by LX2 cells inhibited T-cell proliferation of HCC patients in a dose-dependent manner in vitro. The PI of CD8 + T cells was significantly enhanced following addition of FGL2 antibody to the culture system (LX2/T-cell ratio of 1:10, *p* = 0.002). The level of IFN-γ in mixed cultures was inversely correlated with the number of HSCs and was reversed by incubation with FGL2 blocking antibody.

**Conclusion:**

sFGL2 protein is a novel effector molecule of activated HSCs, which suppresses CD8 + T cell proliferation and interferon-γ production, and it subsequently might contribute to immune suppression during fibrosis and tumorigenesis in the liver.

## Introduction

Hepatocellular carcinoma (HCC) is the fifth most common neoplasm in the world, and the third most common cause of cancer-related deaths [1]. Hepatocellular carcinogenesis is a multifactor, multistep process. The local cellular immune status has been shown to correlate with HCC invasion, recurrence and metastasis [2]. Thus, the HCC immune microenvironment has recently been the focus of extensive research.

Despite their critical functions in the pathogenesis of liver fibrogenesis, hepatic stellate cells (HSCs) have also been shown to possess strong immunomodulatory activities [3–5] and are closely associated with the occurrence and development of HCC [6, 7]. Studies using immune-competent mice and murine hepatic tumor cells revealed that HCCs contain large number of α-SMA-positive HSCs and that co-transplantation of HSCs, through an immunoregulatory form, significantly increases tumor volume [8]. Furthermore, in vitro studies demonstrate that this modulator ability of HSCs is not only associated with a decrease in T-cell infiltration and increased apoptosis of tumor-infiltrating mononuclear cells, but also with the upregulation of Treg cells and B7H1 expression [9]. While the underlying mechanisms controlling this process remain unclear, research in the field of HSC-mediated immunosuppression is becoming increasingly attractive.

FGL2/fibroleukin is a member of the fibrinogen-related protein superfamily and exists in two forms [10]. In tumor and reticuloendothelial cells, FGL2 is expressed as a membrane-associated protein, which acts as a prothrombinase enzyme with the ability to generate thrombin directly from prothrombin [11]. The procoagulant activity of membrane-bound FGL2 (mFGL2) has been implicated in the pathogenesis of several inflammatory disorders [12–18]. However, soluble FGL2 (sFGL2), predominantly secreted by T cells, in particular by Foxp3 + Treg cells [19], has previously been shown to act as an immunomodulator by inhibiting dendritic cell (DC) maturation and T-cell proliferation through the FGL2-FcγRIIB pathway [20, 21]. Recent studies in HCV patients demonstrated that plasma levels of sFGL2 were correlated with the stage of fibrosis and were significantly higher in cirrhotic versus non-cirrhotic patients [22]. Similarly, previous studies in our laboratory also demonstrated that plasma sFGL2 levels were positively correlated with inflammation activity (G1–G3) and fibrosis (S0–S4) of the liver in autoimmune hepatitis patients (unpublished data). Based on this, we propose that sFGL2 is one of several effector factors secreted by HSCs that play a pivotal role in the formation of an immune-tolerant microenvironment in patients with LC and HCC.

In this study, we identify a correlation between unregulated serum sFGL2 levels and HSC activation in HCC patients. Additionally, we confirm that the sFGL2 protein is expressed in the human HSC and LX2 cell lines. Finally, we demonstrate that LX2 cells are capable of suppressing the proliferation and IFN-γ production of CD8 + T cells from HCC patients in vitro via sFGL2. Taken together, these results suggest that targeting FGL2, which contributes to immune suppression during fibrosis and cancer, may be a potential strategy for the prevention and treatment of HCC.

## Materials and methods

### Patients

Forty patients were recruited at Tongji Hospital between March and November 2013 and were classified into four groups: HBV infection, liver cirrhosis (LC), HCC, and HCC with LC. Patients with HCC were recruited from the Department of Hepatobiliary Surgery, while patients with CHB or LC were recruited from the Department of Infectious Diseases. The diagnosis of HCC was confirmed pathologically by either surgical resection of liver tumors or liver biopsies. Subjects were diagnosed with CHB if they tested seropositive for HBsAg and serum aminotransferases were consistently two-fold lower than the upper limit. The diagnosis of cirrhosis was performed in accordance with clinical, biochemical, ultrasonographic and/or histological criteria. The information on the demographics, WBC count, ALT level, TBil, AFP, PT, HBV viral load and anti-viral therapy of all patients is summarized in Table 1. Blood samples were collected from HCC patients before surgery or interventional procedures. None of the cirrhotic patients had undergone splenectomy before. This study was approved by the Ethics Committee of Tongji Hospital.

**Table 1:** Data of patients

Characteristics	CHB (8)	LC (11)	HCC with LC (10)	HCC (11)
Age (years)	43.5 ± 10.3	48.2 ± 11.6	47.3 ± 7.9	45.5 ± 11.7
Gender (M:F)	4:4	5:6	8:2	8:3
WBC count (10^9^/l)	6.2 ± 2.3	6.1 ± 2.2	6.0 ± 2.2	6.3 ± 2.2
ALT (u/l)	38.1 ± 31.1	35.0 ± 48.7	53.1 ± 47.7	41.2 ± 63.0
Tbil (umol/l)	12.6 ± 4.1	21.6 ± 17.5	20.2 ± 19.1	13.1 ± 6.0
PT (s)	13.2 ± 0.7	17.2 ± 1.8	15.1 ± 1.3	13.7 ± 0.9
AFP (ug/l)				
>50,000	0/8	0/11	1/10	1/11
<50,000	8/8 4.1 ± 2.0	11/11 193.9 ± 299.6	9/10 234.7 ± 246.5	10/11 267.2 ± 747.7
HBV-DNA (lg copies/ml)				
<500 copies/ml	4/8	4/11	4/10	5/11
≥500 copies/ml	4/8 3.85 ± 0.35	7/11 4.97 ± 1.52	6/10 4.86 ± 1.49	6/11 3.87 ± 0.67
Antiviral treatment	1(NA)/8	3(NA)/11	3(2NA 1IFN)/10	3(NA)/11
Child-Pugh score		7.7 ± 2.4	6.0 ± 1.0	

The data are presented as the mean ± SD

*WBC* white blood cell, *ALT* alanine transaminase, *Tbil* total bilirubin, *PT* prothrombin time, *AFP* α-fetoprotein

### Blood preparation and isolation of peripheral blood mononuclear cells (PBMCs)

Whole blood was collected from patients and pelleted by centrifugation (1,500×g, 10 min, 21 °C) in heparinized tubes. Plasma was subsequently collected and frozen at −80 °C until analysis. PBMCs were isolated by density gradient centrifugation using Lymphoprep (LymphoPrep™, Norway). Cells were washed twice with RPMI-1640 (Gibco, USA) and resuspended in RPMI-1640 containing 20 % (v/v) fetal bovine serum (FBS) (Gibco, USA) for further analysis.

### Cell culture and Western blot

LX2 cells (kindly provided by Prof. Dr. S. Friedman) were cultured in DEME media containing 10 % (v/v) FBS (Gibco, USA) and penicillin/streptomycin (100 ug/ml, Invitrogen, Carlsbad, CA) at 37 °C in 5 % CO_2_. Membrane and cytosolic fractions were extracted from LX2 cells using a plasma membrane protein extraction kit (Abcam, Cambridge, MA). The expression of FGL2 was assessed by Western blot as previously described [23]. Membranes were first incubated with rabbit anti-Na +/K + ATPase (1:1,000, Santa Cruz Biotechnology, Santa Cruz, CA, USA), mouse anti-Fgl2 (1:400; Abnova, Taiwan, China) and mouse anti-β-actin (1:3,000; Sigma, St Louis, MO, USA) primary antibodies and subsequently with either goat anti-mouse (1:2,500; Bio-Rad Laboratories, Veenendaal, The Netherlands) or goat anti-rabbit (1:5,000; Santa Cruz Biotechnology, CA, USA)-horseradish peroxidase conjugated secondary antibodies. Membranes were incubated with ECL-Plus reagent (Amersham, Piscataway, NJ), and chemiluminescence was detected using BioMax MR Film (Kodak, Rochester, NY).

### Immunofluorescence and flow cytometric staining of FGL2

For immunofluorescence analysis, two cirrhotic liver tissues were fixed for 48 h in 10 % formalin and embedded in paraffin wax before sectioning. Sections of 4 μm thickness for each specimen were prepared on silanized slides. The slides were washed with PBS and then blocked with Protein Block Serum-Free solution. A suspension of LX2 cells (1 × 10^6^ cells/ml) was dripped onto polylysine pre-treated slides and incubated for 10 min. Cells were then fixed with ice-cold acetone for 15 min on ice, then were blocked with 5 % (w/v) bovine serum albumin (BSA). Both slides were incubated with mouse anti-FGL2 [1:250, diluted in PBS containing 1 % (w/v) BSA] overnight at 4 °C, then washed in PBS, incubated with PE-conjugated goat anti-mouse IgG secondary antibody (1:100, diluted in 1 % BSA-PBS, eBioscience, USA) and FITC-conjugated goat anti-human α-SMA antibody (1:100, 1 % BSA in PBS, ebioscience, USA) at room temperature for 1 h. The cells were then washed and stained with propidium iodide (ebioscience, USA) for 10 min. Finally, the cells were washed in PBS and slides were mounted using anti-fade fluorescence glycerin buffer. Cells were visualized by fluorescence microscopy (Olympus IX51, Japan).

For flow cytometric analysis, LX2 cells (1 × 10^6^ cells) were collected in FACS tubes by centrifugation. One set of tubes in group 2 was resuspended in 100 μl of Perm/Wash™ solution (BD, USA) to allow fixation/permeabilization of cells, while operation of rupturing membranes was not used for tubes in group 1. Cells were then incubated with mouse anti-FGL2 antibody (1:100, 1 μg) or normal goat serum as an isotype control at 37 °C for 1 h in the dark. Cells were then washed with 1× Perm/Wash™ solution (1 ml) and incubated with FITC-conjugated goat anti-mouse IgG 1 antibody (1:100) at 37 °C for 30 min in the dark. Cells were washed two times with PBS and resuspended in 300 ul for analysis by a FACSAria Flow Cytometer (BD Biosciences).

### Isolation of T cells from PBMCs

T cells were isolated from PBMCs using the human pan T-cell isolation kit (Miltenyi Biotec, German) and a Midi Macs separator unit, in accordance with the manufacturer’s instructions. In certain experiments, total PBMCs were depleted of non-T cells, and CD3 + T cells were selected.

### Proliferation and functional analysis of CD8 + T Cells

To analyze the PI, CD8 + T cells were labeled with CFSE (Invitrogen, USA) in accordance with the manufacturer’s instructions. Cells were washed with RPMI-1640 supplemented with 10 % (v/v) FBS, and an aliquot of cells was stained with PE-anti-human CD8a (Biolegend, USA). Cells were analyzed by flow cytometry to set the starting fluorescence intensity to that of the parent generation. The remaining cells were seeded in flat-bottomed 96-well plates (1 × 10^6^ cells/well) and cultured with LX2 cells for 5 days in medium containing 20 % (v/v) FBS (as described below). Cells were then stained with PE-anti CD8 antibody and analyzed by flow cytometry. The PI was determined using the Becton-Dickinson ModFit software.

T cells and LX2 cells were cultured at different ratios in medium containing 20 % (v/v) FBS and recombinant IL-2 (2 U/well, R&D Systems, USA) with either anti-CD3 antibody (CD3 stimulation group, 1 pg/ml) or FGL2 antibody (FGL2 blocking group, 5 pg/ml). Negative control cells were cultured without the addition of antibodies (blank group) or IgG2a isotype antibodies (Abcom, USA). Each treatment group was performed in duplicate. After 48 h, culture supernatants were collected for detection of interferon (IFN)-γ levels by ELISA and fresh medium supplemented with stimulants was added for further culturing of cells. After 5 days in culture, cells were collected and CD8 + T-cell proliferation analysis was performed by flow cytometry (BD FACS Canto II).

### Detection of sFGL2 and IFN-γ levels by ELISA

FGL2 levels of patient serum and cell culture supernatants were determined using the LEGEND MAX™ Human FGL2 ELISA Kit in accordance with the manufacturer’s instructions (Biolegend, San Diego, CA, USA). IFN-γ levels in culture supernatants from the CD3 stimulation group, FGL2 blocking group and blank group were detected using an ELISA kit (Dakewe, Beijing).

### Statistical analysis

All graphs were constructed and analysis performed using GraphPad Prism 5 software. Data are expressed as mean ± standard deviation (SD). Differences in serum FGL2 levels between various groups were analyzed by one-way ANOVA or Kruskal-Wallis test. Statistical analyses of CD8 + T-cell proliferation and IFN-γ production were analyzed by paired Student’s *t* test. The level of significance was set at *p* < 0.05.

## Results

### Patient characteristics

The sociodemographic and clinical characteristics of all study groups are displayed as the mean ± SD and are presented in Table 1. No significant differences in the age, gender, white blood cell (WBC) count, alanine transaminase (ALT), total bilirubin (TBil), prothrombin time (PT) or HBV viral load were observed between groups, while no significant differences in the Child-Pugh scores were found in LC patients with or without HCC. Alpha-fetoprotein (AFP) levels were elevated in patients with HCC compared with patients with CHB and LC (*p* = 0.000 for HCC vs. CHB and LC).

### Patient serum sFGL2 levels

**Fig. 1:**
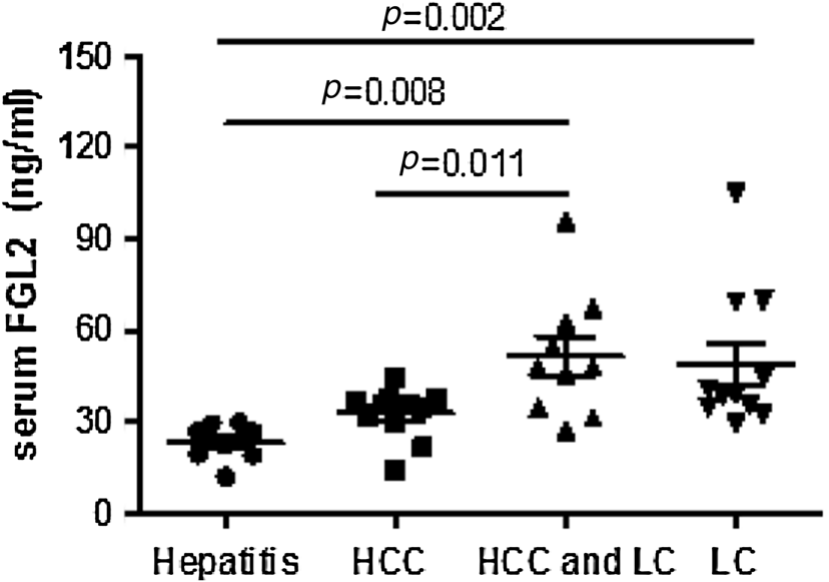
Serum levels of sFGL2 in 8 CHB patients, 11 LC patients and 21 HCC patients (11 with and 10 without LC). Individual values are plotted with mean ± SD shown for each group. Values of *p* are indicated for comparisons of the four groups

The sFGL2 levels in CHB patients (*n* = 8), LC patients (*n* = 11), HCC patients (*n* = 11 with LC, *n* = 10 without LC) are displayed in Fig. 1. Statistical analysis revealed that the serological levels of sFGL2 in HCC and LC patients were significantly higher than in CHB control groups (41.84 ± 17.64 ng/ml/50.61 ± 22.69 ng/ml vs. 23.30 ± 6.19 ng/ml, *p* = 0.0039/*p* = 0.0020). Among HCC patients, the level of sFGL2 in patients with cirrhosis was significantly higher in than those without cirrhosis (51.61 ± 20.30 ng/ml vs. 32.81 ± 8.81 ng/ml, *p* = 0.0108). In contrast, among LC patients, no significant differences in sFGL2 levels were observed between those patients with HCC and non-HCC.

### Expression of FGL2 in liver tissue from cirrhotic patient

**Fig. 2:**
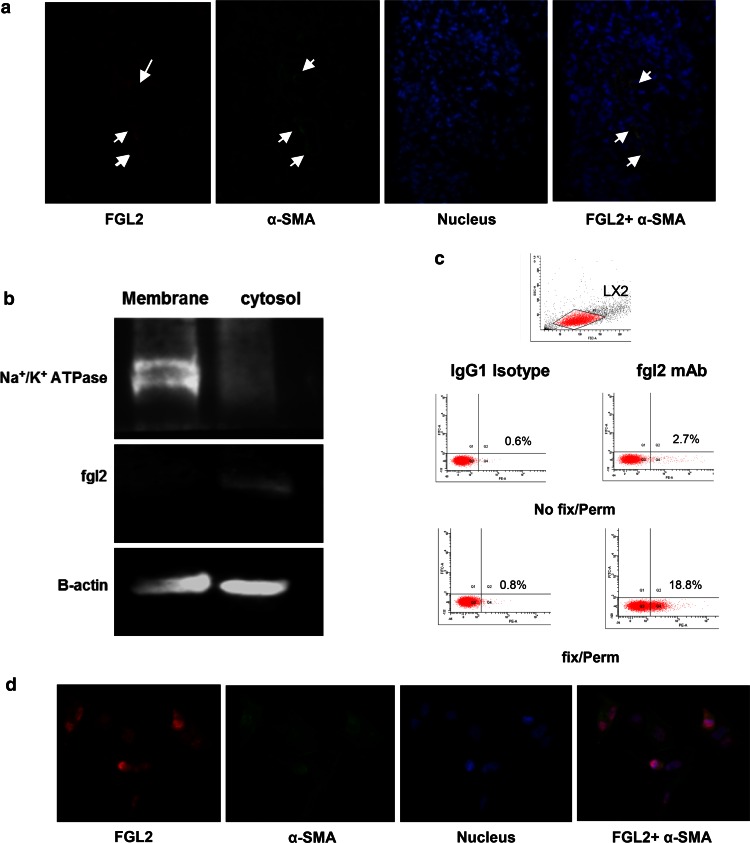
Expression of sFGL2 in liver tissue and human LX2 cells. **a** Double staining immunofluorescence analysis indicates the co-localization of α-SMA (*green*) and FGL2 (*red*). **b** Western blot analysis showing FGL2 expression in cytosol but not membrane fractions. Na + K + ATPase used as a positive control for membrane protein; **b** intracellular staining of FGL2 protein in LX2 cells. FGL2 expression (*upper case*) was detected by flow cytometry after permeabilizationn; **c** double staining for α-SMA (*green*) and FGL2 (*red*). α-SMA-positive expression represents activated HSCs, and sFGL2 is predominantly expressed in the interior of cells. (Color figure online)

A double staining of the immunofluorescence analysis of α-SMA and FGL2 was performed to detect the co-localization of FGL2 and α-SMA in HSCs. α-SMA (Fig. 2a, green) expression was found in sinusoid areas as well as periportal areas, representing a marker of activated HSCs. FGL2 (Fig. 2a, red) could be found at the same area. The merged images indicate the co-localization of FGL2 and α-SMA, revealing that FGL2 was expressed within HSCs.

### Expression of FGL2 in the LX2 cell line

**Fig. 3:**
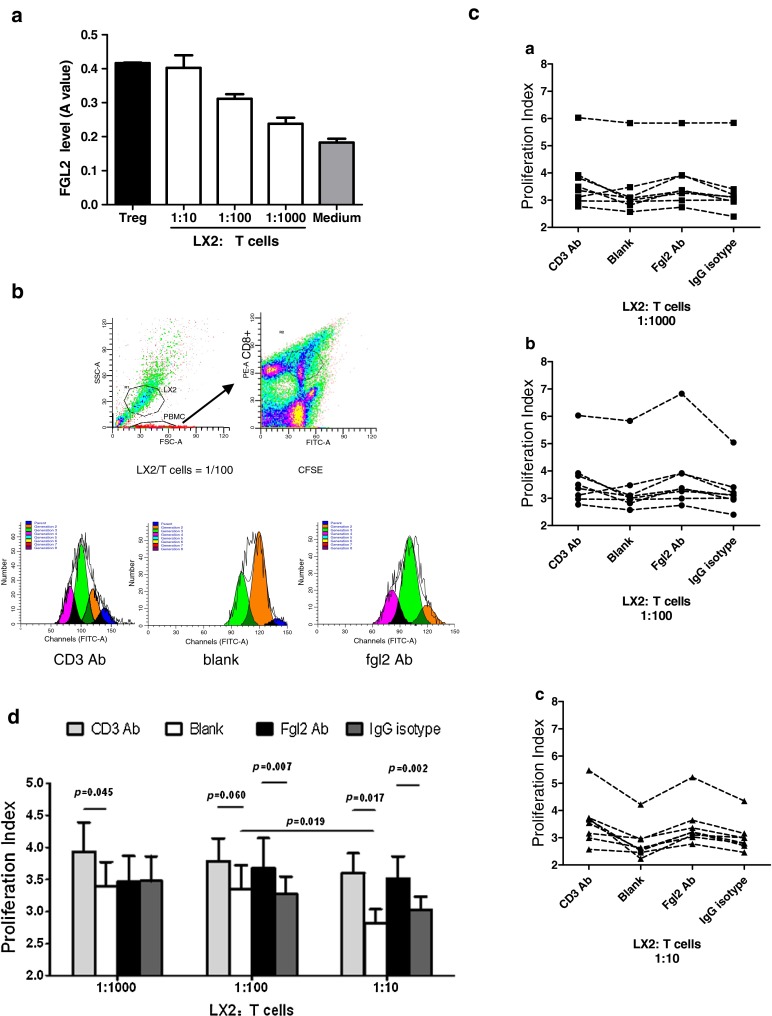
hepatic stellate cells (HSCs) inhibit the proliferation of CD8 + T cells via sFGL2. The T cells isolated from hepatocellular carcinoma (HCC) patients were co-cultured with LX2 cell lines. **a** An increased amount of sFGL2 in the culture supernatant was detected by ELISA as the number of LX2 cells increased. (The supernatant of 1.2 × 106 Treg was used as a positive control, and the culture medium was the negative control.) **b** Flow cytometry analysis of the CD8 + T cell proliferation index; **c** proliferation index of the CD8 + T cells from each sample in CD3 stimulation, FGL2 blocking; blank control groups at different LX2/T-cell ratios. **d** Data are expressed as the mean ± SD. Values of *p* are indicated for comparisons of the four groups

Human LX2 cells were used as a research tool to study the localization of FGL2 in HSC in vitro. Cellular extracts were obtained and membrane and cytosolic fractions were separated using a plasma membrane protein extraction kit. As shown in Fig. 2b, Western blot analysis revealed that FGL2 protein is present in the cellular cytoplasm, but not in the membrane. Intracellular staining for FGL2 identified expression only following permeabilization (Fig. 2c), as assessed by flow cytometry. Furthermore, immunofluorescence analyses revealed that while α-SMA was expressed in both the cytoplasm and the membrane of LX2 cells (Fig. 2d, green), sFGL2 protein was predominantly expressed in the cytoplasm of cells (Fig. 2d, red). These data confirm the constitutive expression of FGL2 in LX2 cells and suggest that sFGL2 exists largely in the soluble form Fig. 3.

### LX2 cells inhibit the proliferation of CD8 + T cells via sFGL2

LX2 cells were co-cultured with T cells at various ratios (1:10–1:1,000) and divided into four groups (CD3 stimulation group, FGL2 blocking group, IgG isotype group and blank group). An increased amount of sFGL2 was evidenced in the culture supernatant as the ratio of LX2 cells to T cells increased (Fig. 2a). After co-culturing for 5 days, the PI CD8 + T cells were analyzed by flow cytometry using the Becton-Dickinson ModFit software (Fig. 2b). The PI of CD8 + T cells from each group is shown in Fig. 2c (a–c). Statistical analysis revealed that the inhibitory effect of LX2 cells on T-cell proliferation was augmented in accordance with the increase in the LX2/T-cell ratio (*p* = 0.019). The PI of CD8 + T cells in the FGL2 blocking group was higher than that in the IgG isotype-treated group at an LX2/T-cell ratio of 1:10 (3.52 ± 0.96 vs. 2.81 ± 0.62, *p* = 0.002) and lower than that in the CD3 stimulation group in all samples (*p* = 0.045 at 1:1,000; *p* = 0.06 at 1:100 and *p* = 0.017 at 1:10) (Fig. 2d).

### LX2 decreases IFN-γ production via sFGL2

**Fig. 4:**
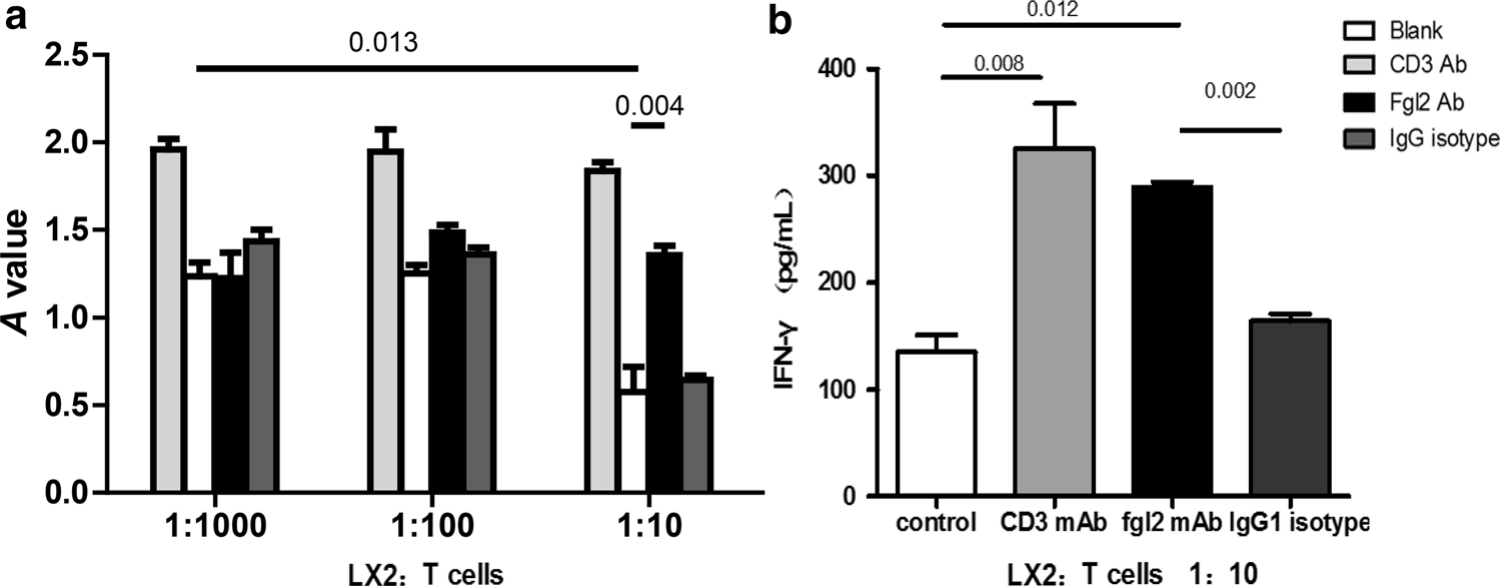
Detection of the IFN-γ level by using ELSIA. The cells were treated and cultured as described in Materials and methods. The supernatants were collected and analyzed. **a** The average A values of IFN-γ in the co-culture mixed supernatants at the T/HSCs ratio varied from 10:1 to 1,000:1. **b** The levels of IFN-γ (pg/ml) detected at a T/HSCs ratio of 100:1. The data are expressed as mean ± SD. Values of *p* are indicated for comparisons of the four groups

IFN-γ levels in culture supernatants were detected by ELISA. The average OD values in each group are shown in Fig. 4a. We observed that the average IFN-γ levels in the control-treated group were lower than those in the CD3 stimulation group in LX2/T-cell mixed cultures (all *p* < 0.001). The OD values in the blank group decreased from 1.57 ± 0.46 at an LX2/T-cell ratio of 1:1,000 and to 0.58 ± 0.21 at an LX2/T-cell ratio of 1:10 (*p* = 0.013). No significant differences in IFN-γ levels were observed between the FGL2 blocking group (1.23 ± 0.36) and control group at an LX2/T-cell ratio of 1:1,000. However, when the ratio of LX2 cells was increased (1:10), the secretion of IFN-γ in the FGL2 blocking group markedly increased compared with IgG isotype-treated cells (0.575 ± 0.145 vs. 1.35 ± 0.145, *p* = 0.004, Fig. 4a) (143.95 ± 33.32 vs. 289.71 ± 12.67 pg/ml, *p* = 0.012, Fig. 4b). In contrast, no significant differences in IFN-γ levels were observed between the FGL2 blocking group and CD3 stimulation group (367.58 ± 54.31 pg/ml, Fig. 4b). These results indicate that HSCs downregulate the secretion of the IFN-γ by T cells, an effect that may be reversed by blocking with anti-FGL2 antibody.

## Discussion

The majority of HCC patients exhibit underlying chronic liver disease and marked impairment in T-cell responses at the baseline level, correlating with tumor burden and poor outcome [24]. LC is the main risk factor underlying the development of HCC, and to date, there is a significant amount of literature highlighting the crosstalk between tumor cells, HSCs and their surrounding microenvironment [25]. Recent reports indicate that HSCs exhibit potent inhibitory activity against the T-cell response that may impair, at least in part, hepatic immune homeostasis and tumor progression in the liver. However, to date, the mechanisms underlying this process remain unclear.

FGL2, which shares 36 % protein homology with the fibrinogen β- and γ-chains, has previously been shown to possess immunomodulatory activity by inhibiting DC maturation and subsequent T-cell proliferation and by polarizing T-helper cell activity toward a type 2 cytokine response [26]. In this study, we demonstrate that patients with cirrhosis express significantly higher levels of FGL2 compared with patients without cirrhosis, independently of whether they have HCC, suggesting that the presence of activated HSCs may account for the high levels of FGL2. No degree of cirrhosis had a positive correlation with the sfgl2 level according to this study, perhaps because of the limited number of patients. However, their correlation has been reported previously by Foerster et al. [27]. The fibrosis stage and FGL2 plasma levels were assessed in 71 out of 80 patients with HCV infection. Plasma FGL2 levels were significantly higher in patients with advanced fibrosis (stage 3–4) compared with patients with mild (stage <1) and moderate fibrosis (stage 2). In addition, analysis of plasma levels of FGL2 according to the activity grade showed a significant difference in patients with higher inflammation as assessed by activity grade (grade >2) compared with those with a lower grade of inflammation (grade <2).

To better demonstrate the correlation of sFGL2 expression with cirrhosis, a double staining of the immunofluorescence analysis of α-SMA and FGL2 in liver specimens from two cirrhotic patient was performed. The merged images indicated the co-localization of FGL2 and α-SMA, revealing that FGL2 was expressed within HSCs. The human LX2 cell line is a well-characterized HSC line that recapitulates many features of the activated HSC [28]. We observed high expression of a soluble form of FGL2 in LX2 cells. HSCs have previously been shown to exhibit features of antigen-presenting cells and to stimulate lymphocyte proliferation [29]. Our study showed an increased amount of sFGL2 in the culture supernatant as the ratio of LX2 cells to T cells increased, and correspondingly, LX2 cells inhibit CD8 + T-cell proliferation in a dose-dependent manner. Blocking sFGL2 with anti-FGL2 antibodies led to an increase in CD8 + T cell proliferation. Moreover, we observed that the level of IFN-γ, which is presumably secreted by CD8 + T cells and reflects the strength of the immune response, is inversely correlated with the number of HSCs and significantly increases in the sFGL2 blocking group in the mixed culture. Since there is no previous evidence to suggest that LX2 cells produce IL-2 [30] or IFN-γ [31], CD8 + T lymphocytes are still considered major sources of IFN-γ in the co-culture system. In our study, the suppressive effect of HSCs was abolished and the secretory level of IFN-γ increased following the addition of FGL2 antibodies to the culture system. These data strongly suggest that sFGL2 secreted by HSCs may be a critical factor underlying the inhibitory effect by suppressing the proliferation of CD8 + T cells.

Multiple mechanisms may be involved in HSC-induced immune tolerance. Jiang et al. [32], observed that HSCs induce apoptosis of conventional CD4 + T cells, primarily via a Fas-FasL-dependent mechanism. A study by Yu MC et al. [33] revealed that quiescent HSCs express low levels of B7-H1. Expression of B7-H1 in HSCs notably increases following exposure to various stimuli, and inhibition of B7-H1 may partially reduce HSC-induced T-cell apoptosis [34]. However, direct contact may not account for the complexity of interactions between HSCs and responder T cells [35]. Our study provides new evidence that sFGL2, as a soluble factor secreted by activated HSCs, represents an additional mechanism accounting for the immune-suppressive effect of these cells. Furthermore, Dangi et al. [36] concluded LPS-stimulated HSCs may promote hepatic tolerogenicity by influencing naturally occurring immunosuppressive CD4 + CD25 + Foxp3 + regulatory T cells, and it has been reported that HSCs could induce CD14 + HLA-DR-/low myeloid-derived suppressor cells from mature peripheral blood monocytes via CD44 [37, 38]. In light of the close relationship between sFGL2 and Treg or DC cells, we propose that HSCs may affect T cells, in addition to other immune cells, via sFGL2. Our previous work [39] demonstrated that there was no significant difference in IFN-γ production of CD8 + T cells when only PBMCs were cultured in the absence or presence of FGL2 Ab, which suggested Treg cells did not significantly regulate CD8 + T cells. Further investigations are required to determine the detailed mechanism of how LX2-expressed FGL2 exerts its effect on CD8 + T cells. It could be either through the FGL2-FcγRIIB pathway directly or by inhibiting the maturation of DC or increasing the function of Treg indirectly.

In conclusion, our study demonstrated the activated HSC cells are capable of secretion of sFGL2. The HSC-produced sFGL2 is a novel functional molecule exerting its negative regulation on CD8 + T cells. This finding provides new insight into understanding the immune response during hepatic fibrosis and tumorigenesis.

## References

[1] Thun MJ (2010). The global burden of cancer: priorities for prevention. Carcinogenesis.

[2] Unitt E (2005). Compromised lymphocytes infiltrate hepatocellular carcinoma: the role of T-regulatory cells. Hepatology.

[3] Winau F (2007). Ito cells are liver-resident antigen-presenting cells for activating T cell responses. Immunity.

[4] Chen CH (2006). In vivo immune modulatory activity of hepatic stellate cells in mice. Hepatology.

[5] Xia YH (2013). T-cell apoptosis induced by intratumoral activated hepatic stellate cells is associated with lung metastasis in hepatocellular carcinoma. Oncol Rep.

[6] Amann T (2009). Activated hepatic stellate cells promote tumorigenicity of hepatocellular carcinoma. Cancer Sci.

[7] Mikula M (2006). Activated hepatic stellate cells induce tumor progression of neoplastic hepatocytes in a TGF-beta dependent fashion. J Cell Physiol.

[8] Zhao W (2012). The role of hepatic stellate cells in the regulation of T-cell function and the promotion of hepatocellular carcinoma. Int J Oncol.

[9] Zhao W (2011). Activated hepatic stellate cells promote hepatocellular carcinoma development in immunocompetent mice. Int J Cancer.

[10] Doolittle RF (1990). The structure and evolution of vertebrate fibrinogen: a comparison of the lamprey and mammalian proteins. Adv Exp Med Biol.

[11] Levy GA (2000). Molecular and functional analysis of the human prothrombinase gene (HFGL2) and its role in viral hepatitis. Am J Pathol.

[12] Marsden PA (2003). The Fgl2/fibroleukin prothrombinase contributes to immunologically mediated thrombosis in experimental and human viral hepatitis. J Clin Invest.

[13] Chan CW (2002). Kinetic analysis of a unique direct prothrombinase, fgl2, and identification of a serine residue critical for the prothrombinase activity. J Immunol.

[14] Ding Y (2010). Expression and significance of fgl2 prothrombinase in cardiac microvascular endothelial cells of rats with type 2 diabetes. J Huazhong Univ Sci Technol Med Sci.

[15] Melnyk MC (2011). The prothrombinase activity of FGL2 contributes to the pathogenesis of experimental arthritis. Scand J Rheumatol.

[16] Chen T (2012). Fgl2 prothrombinase is involved in severe acute pancreatitis-associated liver injury. Hepatogastroenterology.

[17] Liu Y (2012). Downregulation of FGL2/prothrombinase delays HCCLM6 xenograft tumour growth and decreases tumour angiogenesis. Liver Int.

[18] Selzner N (2012). FGL2/fibroleukin mediates hepatic reperfusion injury by induction of sinusoidal endothelial cell and hepatocyte apoptosis in mice. J Hepatol.

[19] Marazzi S (1998). Characterization of human fibroleukin, a fibrinogen-like protein secreted by T lymphocytes. J Immunol.

[20] Chan CW (2003). Soluble fibrinogen-like protein 2/fibroleukin exhibits immunosuppressive properties: suppressing T cell proliferation and inhibiting maturation of bone marrow-derived dendritic cells. J Immunol.

[21] Liu H (2008). The FGL2-FcgammaRIIB pathway: a novel mechanism leading to immunosuppression. Eur J Immunol.

[22] Shalev I (2010). The Role of FGL2 in the Pathogenesis and Treatment of Hepatitis C Virus Infection. Rambam Maimonides Med J.

[23] Gao S (2010). Dual interference with novel genes mfgl2 and mTNFR1 ameliorates murine hepatitis virus type 3-induced fulminant hepatitis in BALB/cJ mice. Hum Gene Ther.

[24] Cabrera R (2010). Hepatocellular carcinoma immunopathogenesis: clinical evidence for global T cell defects and an immunomodulatory role for soluble CD25 (sCD25). Dig Dis Sci.

[25] Yang JD, Nakamura I, Roberts LR (2011). The tumor microenvironment in hepatocellular carcinoma: current status and therapeutic targets. Semin Cancer Biol.

[26] Liu Y (2010). The FGL2/fibroleukin prothrombinase is involved in alveolar macrophage activation in COPD through the MAPK pathway. Biochem Biophys Res Commun.

[27] Foerster K (2010). The novel immunoregulatory molecule FGL2: a potential biomarker for severity of chronic hepatitis C virus infection. J Hepatol.

[28] Xu L (2005). Human hepatic stellate cell lines, LX-1 and LX-2: new tools for analysis of hepatic fibrosis. Gut.

[29] Alisi A, et al. Human hepatic stellate cells are liver-resident antigen-presenting cells. Hepatology. 2011; 54(3):1107; author reply 1108 10.1002/hep.24511 21725990

[30] Mehal WZ (2006). Activation-induced cell death of hepatic stellate cells by the innate immune system. Gastroenterology.

[31] Uemura T, Gandhi CR (2001). Inhibition of DNA synthesis in cultured hepatocytes by endotoxin-conditioned medium of activated stellate cells is transforming growth factor-beta and nitric oxide-independent. Br J Pharmacol.

[32] Jiang Z (2013). Hepatic stellate cells promote immunotolerance following orthotopic liver transplantation in rats via induction of T cell apoptosis and regulation of Th2/Th3-like cell cytokine production. Exp Ther Med.

[33] Yu MC (2004). Inhibition of T-cell responses by hepatic stellate cells via B7-H1-mediated T-cell apoptosis in mice. Hepatology.

[34] Gu X (2013). Interferon- gamma triggers hepatic stellate cell-mediated immune regulation through MEK/ERK signaling pathway. Clin Dev Immunol.

[35] Chen CH (2010). Cotransplantation of hepatic stellate cells attenuates the severity of graft-versus-host disease. Transplant Proc.

[36] Dangi A (2012). Selective expansion of allogeneic regulatory T cells by hepatic stellate cells: role of endotoxin and implications for allograft tolerance. J Immunol.

[37] Hochst B (2013). Activated human hepatic stellate cells induce myeloid derived suppressor cells from peripheral blood monocytes in a CD44-dependent fashion. J Hepatol.

[38] Chou HS (2011). Hepatic stellate cells regulate immune response by way of induction of myeloid suppressor cells in mice. Hepatology.

[39] Xu L (2012). Inhibitory function of Tregs via soluble FGL2 in chronic hepatitis B. J Huazhong Univ Sci Technol Med Sci.

